# Implementing an Interdisciplinary Committee to Reduce Psychotropic Drug and Increase Behavioral Intervention Use

**DOI:** 10.1093/geroni/igaa057.980

**Published:** 2020-12-16

**Authors:** Lauren Hess Conrad, David Portman

**Affiliations:** 1 Butler VA Health Care System, Pittsburgh, Pennsylvania, United States; 2 VA Butler Healthcare System, Butler, Pennsylvania, United States

## Abstract

In Fiscal Year (FY) 2018, the Butler VA Health Care System’s Psychotropic Medication and Behavior Management Committee was identified as a Veterans Integrated Service Network 4 Best Practice. The goal of this committee is to reduce unnecessary psychotropic medication use and polypharmacy and to increase behavioral intervention implementation among Community Living Center (CLC) Veterans. This committee meets quarterly to review Psychotropic Drug Safety Initiative data, behaviors and behavior care plans, and all psychotropic medications prescribed to Veterans. Psychiatric diagnoses, changes to psychotropic medications, and appropriate behavioral interventions are discussed. Committee members take responsibility for action items in accordance with their discipline; documentation of recommendations are made in quarterly behavioral health assessments in CPRS; and follow-up on action items is completed at twice weekly interdisciplinary treatment team meetings, weekly behavior rounds, and/or as needed. From the first quarter (Q1) of FY16 to Q1 FY20, the Butler VA CLC has seen decreased prescriptions of 2 or more anticholinergics (6.6% to 0.80%), antihistamines (12.5% to 5.9%), benzodiazepines (24.7% to 11.0%), and benzodiazepines or sedative hypnotics (23.2% to 9.0%). While prescription of antipsychotic use has increased (Q1 FY20 = 23.8%), the committee will follow Long Term Care Institute guidelines for gradual dose reductions, behavioral interventions, and as needed psychotropic medication PRN use. This committee provides an interdisciplinary forum to discuss and implement beneficial changes to pharmacological and non-pharmacological interventions among all CLC Veterans. The committee is a valuable process for monitoring and reinforcing best practices that may be easily replicated across VA CLCs nationwide.

